# Self-healing capacity of fiber-reinforced calcium phosphate cements

**DOI:** 10.1038/s41598-020-66207-2

**Published:** 2020-06-10

**Authors:** Anne V. Boehm, Susanne Meininger, Uwe Gbureck, Frank A. Müller

**Affiliations:** 1 Otto Schott Institute of Materials Research (OSIM), Friedrich Schiller University Jena, Löbdergraben 32, 07743 Jena, Germany; 2 Department for Functional Materials in Medicine and Dentistry (FMZ), University of Würzburg, Pleicherwall 2, 97070 Würzburg, Germany

**Keywords:** Health care, Materials science

## Abstract

A major problem concerning the mechanical properties of calcium phosphate cements (CPC) is related to their inherent brittleness, which limits their applicability to non-load bearing bone defects. In this work the preparation of a damage tolerant CPC is presented, where the incorporation of functionalized carbon fibers facilitates steady state flat crack propagation with crack openings below 10 µm. A subsequent self-healing process in simulated body fluid, that mimics the *in vivo* mineralization of bioactive surfaces, closes the cracks and completely restores the mechanical properties. Hereby, two pathways of self-healing are presented: i) intrinsic healing that bases on the inherent bioactive properties of the cement matrix and chemically treated fibers, and ii) capsule based extrinsic healing, where H_2_PO_4_^-^ is released as an initiator for the apatite formation. Such damage tolerant CPCs with self-healing capacity are of particular interest to increase the lifetime of implants as well as in the field of load-bearing bioceramics.

## Introduction

In millions of years, evolution developed materials with self-healing properties. These natural materials have the capability, to partially or completely restore in shape or function after the event of damage^1^. In nature there are many familiar examples of self-healing that we take for granted, such as spontaneous healing of ruptured skin or mending of broken bones. Healthy human bone is a material that is permanently in change^2^. Cells, osteoclasts and osteoblasts, respond to biomechanical stimuli and degrade or build bone accordingly, by using ions, such as calcium and phosphate that are present in the blood plasma as well as stored in already existing bones and teeth^3^. Due to these actions, a broken bone is able to mend.

In the recent 20 years, intense research on the development of self-healing materials was performed^1,4,5^. Hereby, several strategies are to be distinguished. First of all, a distinction is made between autonomic healing, where no external trigger or action is required and non-autonomic healing, that presupposes external triggers. Furthermore, a differentiation is made between intrinsic healing, based on the inherent properties of the material, and extrinsic healing using external agents supplied in, for example, capsule systems^6^. Since polymers show chain mobility even at comparatively low temperatures, and a large number of chemical modification options are available, a multitude of self-healing mechanisms are found for this by far most intensively studied material class^4,5,7–13^. Nevertheless, pathways for metals, ceramics and concretes were developed, as well^12,14–16^. Many healing methods require external triggers, with temperature regimes being the most utilized^5^. In terms of extrinsic healing with capsule systems, the repeatability of the healing process is restricted^1^. These factors can limit the fields of potential applications. However, applications with poor accessibility, for example in the construction industry or especially in clinical practice, particularly depend on self-healing materials, since here the replacement or even the detection of defects can be problematical. For clinical practice, numerous self-healing polymeric biomaterials and hydrogels were investigated and developed in the past 10 years^9,13,17^. For brittle materials like cements or concrete the stabilization of the defect is essential for the healing strategy and can be achieved by different pathways^18^. In the case of concretes utilized for construction some systems with self-healing capability were developed. Hereby, different strategies were investigated with re-hydration, capsule systems or biological processes being most prominent^12^. For the latter, bacteria are added to the cement system, which produce mineralization products closing the crack^19^. Defect stabilization and healing itself is typically based on the formation of salts in the crack, blocking of cracks with impurities or particles resulting from water infiltration or crack spalling, further hydration of unreacted cement or finally the expansion of cementitious matrix in the crack flanks^15^. Self-healing with apatite materials was shown for apatite coatings and by utilizing phosphate-containing hydrogel in Portland cement paste^20,21^.

To design a self-healing calcium phosphate cement (CPC) we suggest a mechanism with inspiration from self-healing mechanisms used for construction materials, utilizing crack stabilization and closing by damage tolerance and subsequent the formation of precipitate in the crack. Although both cements in the construction industry and CPCs, have to struggle with their brittle behavior and their poor accessibility in application in terms of self-healing, there are some very important differences in the challenge of designing a self-healing material. Besides others, the size of the components and workpiece differ and the choice of materials *in vivo* is limited to biocompatible ones. In general, CPCs result from the setting reaction of a calcium phosphate (CaP) powder and a liquid phase. Depending on the pH of the reaction either apatite or brushite is formed. However, in contrast to polymeric biomaterials or hydrogels this implant material, in clinical application since 1994 and under intense research for roughly 35 years by now, has significant advantages^22–25^. Due to the paste-like state of CPC between mixing and setting, the cement can be injected and is therefore of particular interest in minimal invasive surgery^25^. Beyond, the paste is capable to fill complex geometries and to ensure an intimate bone-implant-contact. Setting itself is an autonomous process, at moderate temperatures and without significant volume changes, which occurs *in vivo*^26^. Thus, under physiological conditions the resulting precipitate, shows high chemical and structural similarity to mammalian hard tissue, that is composed of biological apatite^27^. Finally, setting reactions at physiological temperatures allows the incorporation of drugs such as antibiotics, anti-inflammatory agents or growth factors in order to stimulate certain biological reactions^28^. Beyond these advantages, however, CPCs are suffering from their inherent brittleness, which restricts their application^29^. To overcome this drawback, damage tolerant systems were developed using, e.g. fiber reinforcement^30–34^. Among others, carbon fibers (C-fiber) proved to be effective^35–37^. In a previous study we investigated the influence of chemical fiber treatment on the mechanical performance of CPC^35^. The bending strength of CPC was increased from 9 MPa to 19 MPa by adding 1 wt% untreated C-fibers. A chemical pretreatment of the C-fibers with *aqua regia* and calcium chloride lead to a further increase to 30 MPa. Additionally, the opening of occurring cracks was stabilized and fatal crack propagation was prevented, resulting in enhanced Work of fracture *(WOF5*). In addition to the tensile strength of the C-fibers, the surface chemistry of the fibers also influences the setting reaction of the CPC and thus the resulting matrix structure and strength. In the case of CPC with modified fibers, the fiber surface treatment increases the matrix fiber bond, leading to adhered matrix material that remains on the fibers surface after fracture. These remaining matrix particles impede the fiber pull-out, since they cause higher friction. Consequently, higher strength and *WOF5* is observed for the composite^35^.

In the present study, we go even further and present a self-setting, damage tolerant CPC with *in vitro* self-healing capability. For this purpose, an apatite cement with C-fiber reinforcement was damaged in a controlled manner and healed in simulated body fluid (SBF). Since SBF mimics the composition of the inorganic part of human blood plasma, it can reproduce the *in vivo* formation of apatite on bioactive surfaces and is therefore used to investigate the *in vitro* bioactivity^38–40^. In this context, a surface is considered to be bioactive, if it facilitates the heterogeneous nucleation and growth of apatite^41,42^. Combining the *in vitro* ability of SBF to form apatite with a damage-tolerant bioactive material, the aim of the present study is to design a cement-based implant material with an intrinsic self-healing capacity, where cracks are instantly healed by a biomimetic mineralization process. Beyond, also an extrinsic healing pathway will be investigated, using H_2_PO_4_^-^ supplying capsules, which trigger the precipitation of CaP. Such self-healing CPC are of potential interest for load-bearing orthopedic applications, since occurring cracks might be filled with new bone instead of leading to catastrophic *in vivo* failure.

## Materials and Methods

**Figure 1:**
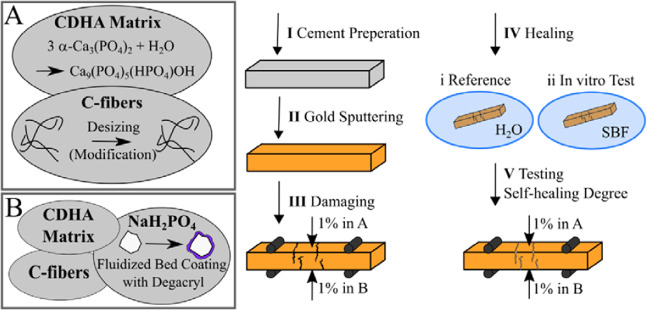
Sample preparation and regime of self-healing tests. The left part illustrates the two examined cement systems, (**A**) calcium deficient hydroxyapatite (CDHA) matrix and C-fibers as well as (**B**) CDHA matrix, C-fibers and NaH_2_PO_4_ supply, whereas on the right the procedure for self-healing tests with the steps I to V is schematically visualized.

The experimental procedure for sample preparation and self-healing tests is schematically illustrated in Fig. 1. As indicated on the left, the CPC samples were prepared as a composite of C-fibers embedded in an α-tricalcium phosphate (*α*-TCP) matrix. Partly sodium dihydrogen phosphate (NaH_2_PO_4_) capsules were added to enable extrinsic healing experiments. Besides other tests, self-healing was examined regarding to the regime presented on the right in Fig. 1. In the following chapters, the single parts of the experimental procedure are described in detail.

### Cement preparation

*α*-TCP was prepared by sintering calcium hydrogen phosphate (CaHPO_4_, Mallinckrodt-Baker, Germany) and calcium carbonate (CaCO_3_, Merck, Germany) in a molar ratio of 2:1 for 5 h at 1400 °C followed by quenching to room temperature. The sintered cake was crushed and passed through a 125 μm sieve followed by ball milling at 200 rpm for 4 h^43^. A solution of 1 M trisodium citrate (Na_3_C_6_H_5_O_7_, Carl Roth, Deutschland) and 2.5 wt% disodium hydrogen phosphate (Na_2_HPO_4_, Carl Roth, Deutschland), prepared with demineralized water, served as liquid phase in the cement preparation.

C-fibers (Nippon Graphite Fiber Corporation, Japan) with a diameter of *d* = 7 µm were cut to a length of 1 cm before desizing twice in boiling isopropanol (Carl Roth, Germany). Subsequently, the fibers were dried in a freeze dryer and processed as they are or stirred for 40 min in *aqua regia* (HCl, Carl Roth, Germany and HNO_3_, Carl Roth, Germany), washed with water and stored in 1 M calcium chloride (CaCl_2_, VWR Chemicals, Belgium) solution for 1 day, as reported in detail previously^35^. After the modification the C-fibers were freeze-dried.

To prepare capsules NaH_2_PO_4_·2 H_2_O (VWR Chemicals, Belgium) salt was coated with Degacryl (PMMA, 15 kDa, Evonik Industries AG, Germany) dissolved in acetone using a fluidized bed coating (Mini Glatt 12104, Glatt, Germany). Particle size and surface ratio were estimated by analyzing light microscopic images of NaH_2_PO_4_ capsules and cement fracture surfaces using the software ImageJ. Light microscopy (Axio Vision, Carl-Zeiss, Germany) was  performed in light field using z-stack.

*α*-TP, 1 wt% C-fibers, and optionally 2 wt% NaH_2_PO_4_, according to the compositions listed in Table 1, were mixed in dry state by hand and combined with the liquid phase at a powder-to-liquid ratio (PLR) of 3 g∙mL^−1^. The mixed CPC pastes were transferred into silicon molds and stored at high humidity and 37 °C for 4 h. Then they were stored to demineralized water for final setting at 37 °C for 7 days. Hereby, 40 g *α*-TCP and the corresponding amounts of additives were used to obtain 32 samples, 16 of which were each tested with similar parameters (e.g. 16 reference in water and 16 immersion in SBF for specific time). After setting in demineralized water for 7 days, CPCs were ground plane-parallel using abrasive paper up to a grit of 1000.

**Table 1:** Sample composition and nomenclature.

Sample	Fiber Additive	Capsule Additive
CPC	—	—
C-CPC	1 wt% untreated C-fibers	—
C_m_-CPC	1 wt% *aqua regia* and CaCl_2_ treated C-fibers	—
PO_4_-C-CPC	1 wt% untreated C-fibers	2 wt% NaH_2_PO_4_ capsules
PO_4_-C_m_-CPC	1 wt% *aqua regia* and CaCl_2_ treated C-fibers	2 wt% NaH_2_PO_4_ capsules

Applying the Gilmore needle test, the initial (needle diameter 2.12 mm, weight 113 g) and final (needle diameter 1.06 mm, weight 454 g) setting time was estimated using plate specimens with a diameter of 20 mm.

### Mechanical properties and self-healing capacity

The mechanical properties of CPC were characterized using a universal testing machine (Z020, Zwick, Germany). In three-point bending tests specimens with dimensions of length *l* = 30 mm, height *h* = 3–4 mm and breadth *b* = 6 mm were tested applying a support span of *L* =  20 mm, a loading rate of 1 mm·min^−1^_,_ and a preload of 0.1 N. The bending stress *σ*_*b*_ was calculated according to Eq. 1, with the force *F*. The strain *ε*_*b*_ was calculated according to Eq. 2 with the  deflection *s*. Additionally, the Work of fracture up to a strain of 5% *WOF5* was calculated according to Eq. 3. Hereby the force *F* was integrated over the deflection *s* from *s*_0_ = 0 to the deflection *s*_5_, where *ε*_*b*_ equals 5%. Although samples show much higher strain at failure, the residual strength at higher strain strongly depends on fiber orientation, causing the error  being vast. Since 5% strain is closer to the actual strain of bone^44^ this limit was chosen for a better comparability also concerning clinical applications.

1 $${\sigma }_{b}=\frac{3FL}{2b{h}^{2}}$$

2 $${\varepsilon }_{b}=\frac{6sh}{{L}^{2}}\cdot 100 \% $$

3 $$WOF5=\frac{{\int }_{{s}_{0}}^{{s}_{5}}Fds}{bh}$$

For the test of the self-healing capacity the steps I to V, shown in Fig. 1 were applied. Hereby each test was performed with *n* = 16 specimens. (I) Samples were prepared according to the cement preparation procedure and dried after preparation. (II) Cement bars were sputtered with gold, which is bio-inert, to ensures that a mineralization is directed to freshly formed defects. (III) Subsequently, the specimens were damaged in a controlled manner by measuring the bending stress up to an elongation of 1% in both directions A and B one after the other by turning the sample. 1% strain was chosen, since the strain at maximum bending strength is in this range and a reduction of strength upon second loading without healing is measurable. (IV) Samples were stored for 7 days in either SBF or demineralized water at 37 °C. SBF solutions with ionic concentrations almost equal to the inorganic part of human blood plasma were prepared according to Müller *et al*.^38^ Hereby the immersion volume was 200 mL. (V) After rinsing with demineralized water and drying, the bending stress up to a strain of 1% was measured in both directions in the same order as previously. The degree of self-healing *SHD* is defined as the sum of strengths in both directions A and B after storage *σ*_bA_^*^ and *σ*_bB_^*^, divided by the sum of strengths before healing *σ*_*bA*_ and *σ*_*bB*_, Eq. 4.

4 $$SHD=\frac{({\sigma }_{bA}^{\ast }+\,{\sigma }_{bB}^{\ast })}{({\sigma }_{bA}+\,{\sigma }_{bB})}\cdot 100{\rm{ \% }}$$

Healing kinetics were measured mechanically, applying the above described regime for healing periods of 1, 3, 5 and 7 days, respectively. Beyond, the composition of SBF was measured everyday by taking 5 mL from the solution. 5 mL of 20 vol% HNO_3_ were added and the solution was passed through a syringe filter with a pore size of 0.45 µm. The concentration of calcium and phosphor was quantified using a simultaneous radial ICP-OES spectrometer 725ES (Agilent, Waldbronn, Germany) with a CCD-detector. For these tests the immersion volume was raised to 250 mL without further refilling. On the basis of the results of the kinetic measurements, cyclic healing in SBF was performed using a healing period of 5 days for three cycles by repeating steps III to V. Scanning electron microscopy (SEM, Sigma VP, Carl-Zeiss, Germany) on reinforced and healed CPC was performed after 7 days of (I) setting or (V) healing and subsequent drying.

### Statistical analysis

The self-healing capacity in terms of *SHD* was analyzed for significant differences between groups by one-way ANOVA using both the comparison with the respective reference in water and the differences between the healed samples. For all tests the level of significance was set at p < 0.05.

## Results

### Cement characteristics and mechanical properties of CPC

**Figure 2:**
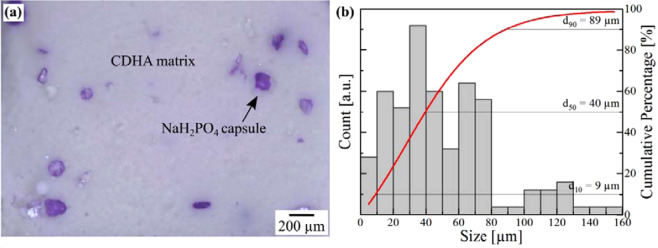
(**a**) Fracture surface of PO_4_-CPC showing capsules distribution as well as (**b**) size distribution of NaH_2_PO_4_ capsules.

In this study, the self-healing of cements reinforced with C-fibers and to which additional NaH_2_PO_4_ capsules were optionally added was investigated. Whereas the properties of C-fibers reinforced CPC were already published in a previous study^35^, the characteristics of the capsules and their influence are reported in the following. The capsules consist of a NaH_2_PO_4_ salt, which was coated with the purple stained polymer Degacryl, which is based on PMMA. The capsules are evenly distributed in the cement and have a surface ratio of 7% ± 3% at the fracture surface (Fig. 2a). The size distribution of the irregularly shaped NaH_2_PO_4_ capsules displays a broad distribution with a *d*_50_ of 40 µm (Fig. 2b).

**Figure 3:**
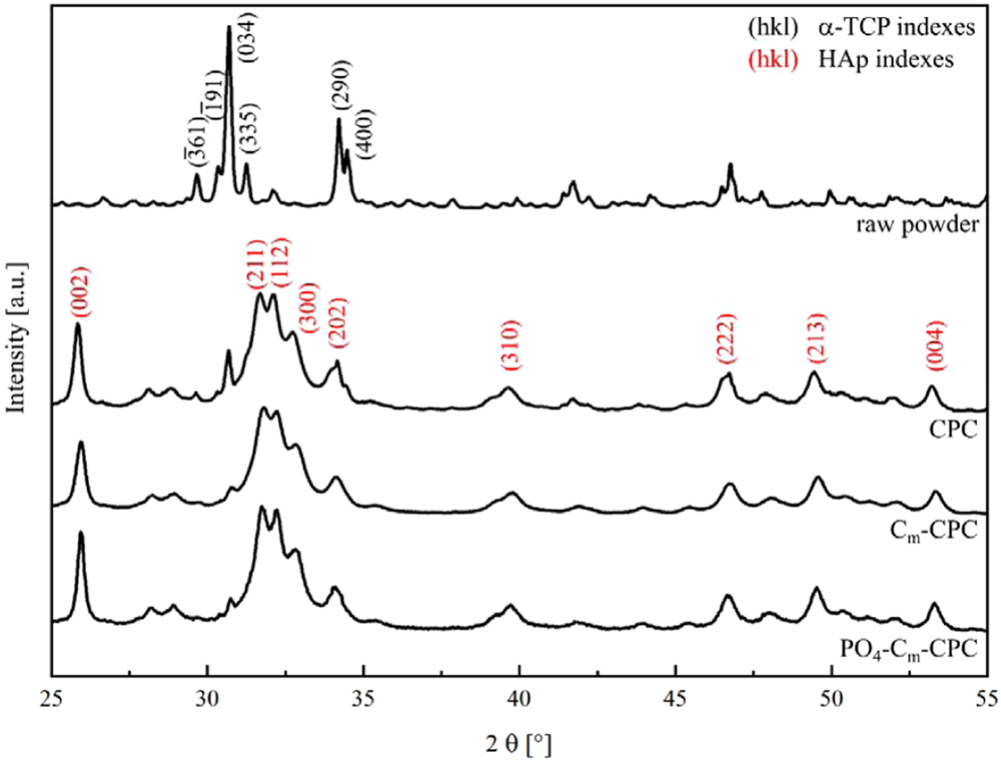
X-ray diffraction analyses of the raw powder, CPC, C_m_-CPC and PO_4_-C_m_-CPC after 1 week setting in demineralized water.

The X-ray diffraction in Fig. 3 shows, that in all systems a cement matrix consisting of calcium deficient hydroxyapatite (CDHA) is formed in a setting process from the raw powder *α*-TCP and an aqueous solution. The diffraction patterns show the presence of two phases, Hydroxyapatite (HAp) and α-TCP. The Rietveld analyses presented in Table 2 revealed that for CPC without additives the content of α-TCP is highest at 13%, while the addition of both fibers and NaH_2_PO_4_ capsules leads to a reduction of the α-TCP content to 9% and 5%, respectively.

**Table 2:** Phase quantification from XRD using Rietveld.

Sample	α-TCP [%]	HAp [%]	Rwp
α-TCP	100	—	4.612
CPC	13	87	8.067
C_m_-CPC	9	91	8.043
PO_4_-C_m_-CPC	5	95	8.800

**Figure 4:**
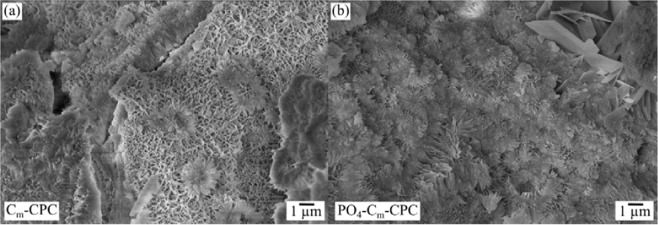
Scanning electron microscopy of fracture surfaces, showing (**a**) C_m_-CPC and (**b**) PO_4_-C_m_-CPC.

Figure 4a illustrates the resulting morphology of the CPC matrix without further additives. It consists of CDHA crystals, which form a porous network. The initial and final setting time for pure CPC was estimated as *t*_i_ = 25 min and *t*_f_ = 105 min and shows upon fiber addition a significant reduction^35^. This effect was even more pronounced, when PO_4_^3-^ capsules were added in the PO_4_-C_m_-CPC system, where the setting times are reduced to *t*_i_ = 13 min and *t*_f_ = 21 min. Beyond, the addition of capsules led to a smaller crystal size (Fig. 4b).

During the mixture, some of the capsules tear apart and release H_2_PO_4_^-^, causing a higher local saturation and consequently an accelerated nucleation. Thus, the crystal size is reduced, also resulting in altered mechanical behavior (Table 3). The incorporation of capsules increased the bending strength by 100% when untreated fibers were used and by 50% when chemically pretreated fibers were used. Besides the increase of strength, all reinforced cements show enhanced damage tolerance in comparison to pure CPC. Hereby, the *WOF5* was enhanced from 0.02 kJ·m^−2^ for pure CPC to 1.9 kJ·m^−2^ in the case of C_m_-CPC^35^. Upon the addition of PO_4_-supply, *WOF5* remained in the same range with *WOF5* = 1.7 kJ·m^−2^ for both, PO_4_-C-CPC and PO_4_-C_m_-CPC.

**Table 3:** Setting and mechanical properties of CPCs as well as *SHD* after immersion for 7 days in water. Listed values are mean ± standard variation.

Sample	Initial Setting Time [min]	Final Setting Time [min]	Bending Strength [MPa]	Work of Fracture to ε_b_ 5% [kJ·m^−2^]	Degree of Self-Healing in SBF [%]
CPC	25.0 ± 0.5	105 ± 5	9.2 ± 1.7	0.02 ± 0.004	0
C-CPC	20.0 ± 0.5	65 ± 2	19.3 ± 1.9	0.7 ± 0.4	102 ± 18
C_m_-CPC	18.5 ± 0.5	50 ± 2	30.3 ± 2.8	1.9 ± 0.3	107 ± 55
PO_4_-C-CPC	16.0 ± 0.5	25 ± 1	37.8 ± 4.2	1.7 ± 0.6	110 ± 17
PO_4_-C_m_-CPC	13.0 ± 0.5	21 ± 1	45.0 ± 3.8	1.7 ± 0.5	110 ± 8

Data of setting and mechanical properties of CPC, C-CPC and C_m_-CPC are captured from previous study^35^.

**Figure 5:**
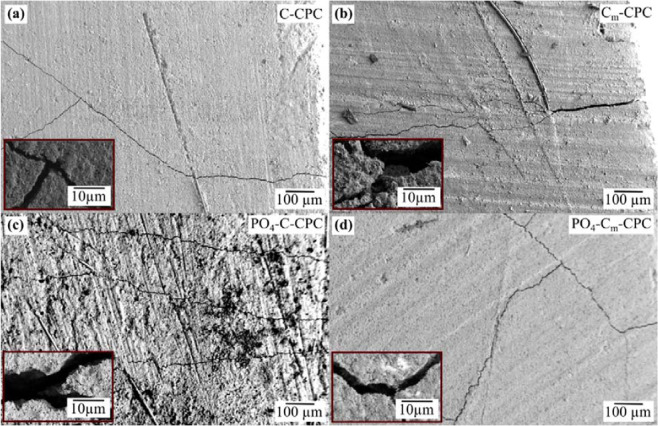
Crack propagation and detailed view after 3-point bending of (**a**) C-CPC, (**b**) C_m_-CPC, (**c**) PO_4_-C-CPC and (**d**) PO_4_-C_m_-CPC. Scanning electron microscopy was performed on the lateral surfaces between the pressure points.

The damage tolerance of the reinforced systems is caused by changes in the cracking behavior (Fig. 5). Whereas for CPC only one single crack with a fatal crack opening propagation is observed, the fiber reinforcement leads to multiple cracking and a crack stabilization with crack openings below 10 µm. Thus, the damage tolerance can be assumed for all fiber reinforced CPC-systems shown in this study.

### Self-healing capacity

**Figure 6:**
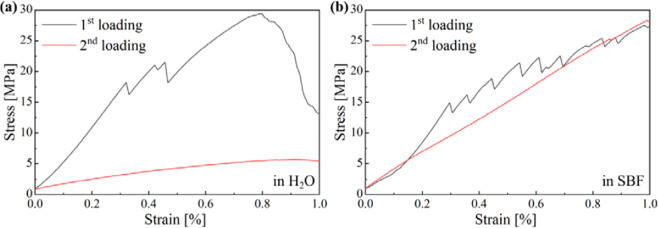
Stress-strain curves of C_m_-CPC showing first loading (black) and second loading (red) during self-healing tests for samples stored 7 days in (**a**) water^-^ and (**b**) SBF.

The self-healing capacity of cements was proven by the mineralization-induced closure of cracks and a recovery of their mechanical properties, respectively. The comparison of the stress-strain curves for CPCs prior and after immersion shows self-healing for all samples in SBF and for CPCs with capsules also in water. Since the bending tests were applied only up to a strain of 1% and in both directions, the individual measurements are not significant for the mechanical properties of the material, but the relation of strength from first measurement to second measurements reflects the self-healing capability of the CPCs. Exemplarily, stress-strain curves for C_m_-CPC at first and second loading stored for 7 days in water and SBF, respectively, are shown in Fig. 6. Hereby, not only the maximum strength, but also the similar slope in the case of samples stored in SBF implicates the self-healing of CPCs. However, while individual maxima are visible at the first load, the stress increases more continuously with strain at the second load.

**Figure 7:**
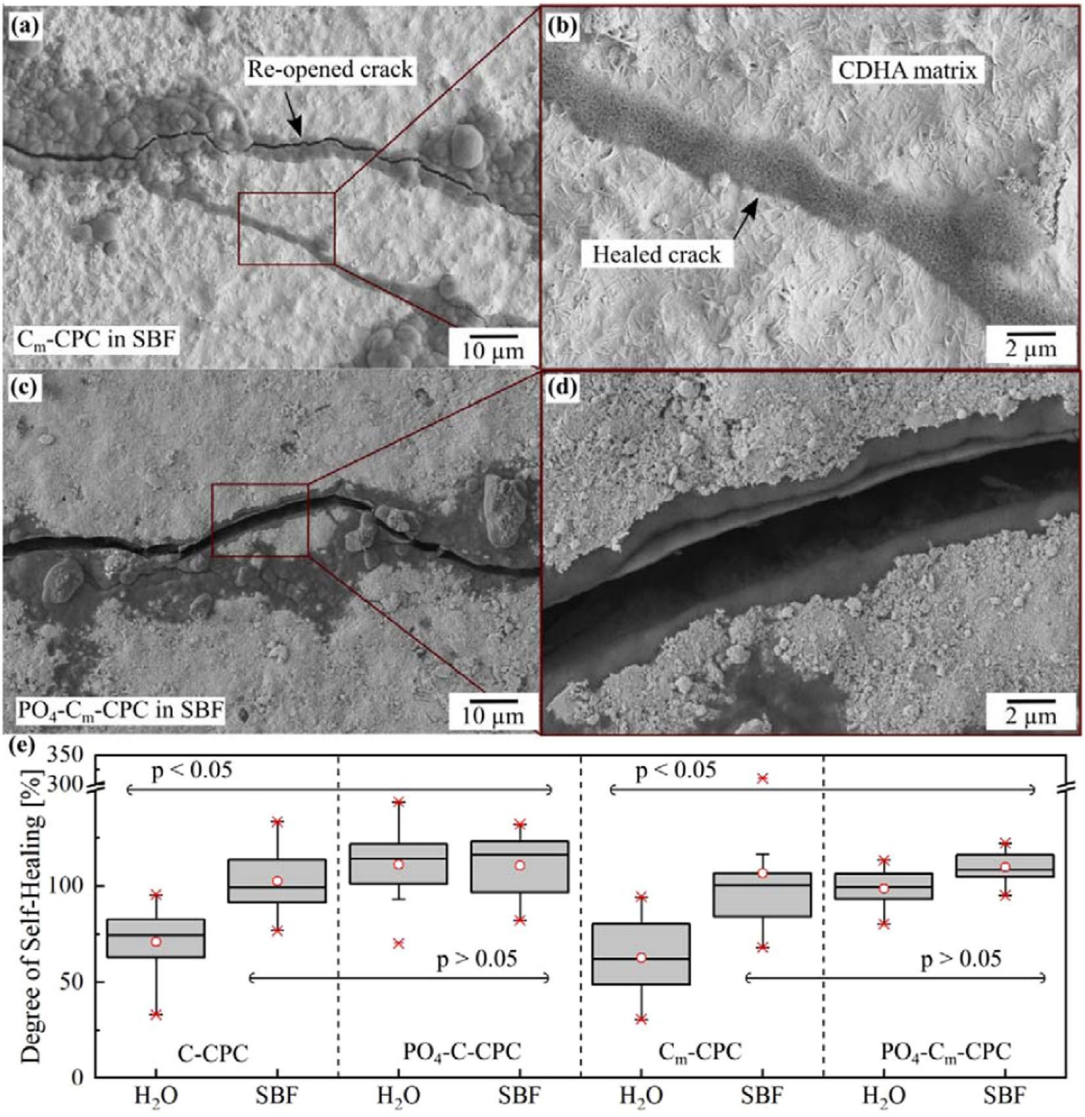
SEM images of a C_m_-CPC that was damaged, healed and again damaged in (**a**) overview and (**b**) a detailed view of the mineralization in the crack, as well as for PO_4_-C_m_-CPC with similar regime in (**c**) and (**d**). Scanning electron microscopy was performed on the side surfaces between the loading bars. Self-Healing capacity in terms of mechanical properties after 7 days storage in water or SBF of (**e**) C-CPC as well as PO_4_-C-CPC, and C_m_-CPC as well as PO_4_-C_m_-CPC. Results from ANOVA are indicated with p </> 0.05.

Figure 7a shows the SEM image of a C_m_-CPC after self-healing, reflecting the damaging-healing-damaging process. In composites consisting of a CDHA matrix (light grey) reinforced with chemically treated C-fibers, defects were introduced by straining. These defects healed during exposure to SBF by a mineralization within the crack. The mineralization product can be   distinguished by its darker grey color. In the detailed view of the healed crack a porous plate-like morphology of the precipitate can be observed (Fig. 7b). It can also be seen that the crack propagating from the left splits into two, both of which are mineralized. When the strength was examined again after self-healing, the healed crack is reopened. Contrary to the previous course, this time, however, the crack did not split, but ran exclusively along the upper line. A similar cracking pattern can be observed in the case of PO_4_-C_m_-CPC shown in Fig. 7c,d. For this particular crack, which was formed during the first loading, the closure occurred during immersion in SBF. At the second loading, the crack was reopened, with the crack running through the mineralized crack filling. Figure 7e quantifies the self-healing capacity of C-CPC, PO_4_-C-CPC, C_m_-CPC and PO_4_-C_m_-CPC. In general, both plots show a similar course. However, as mentioned earlier, but not obvious in the boxplots due to the normalization in the formula of the self-healing degree *SHD*, the absolute strength of the cements strongly differs. Damaged C-CPCs did not recover when stored in water but reached only 60–80% of their original strength (Fig. 7e). On the contrary, when analogous C-CPCs were stored in SBF an average *SHD* of 102% was observed. A similar increase in *SHD* was also detected for C_m_-CPC (Fig. 7e). Here, the reference in water shows about 63% in the second test of the mechanical properties, while the healing in SBF causes an average restoration of *SHD* of 106%. The addition of capsules changed the healing behavior particularly in water, where *SHD* reached 110% and 98% for untreated and modified fibers, respectively (Fig. 7e).

**Figure 8:**
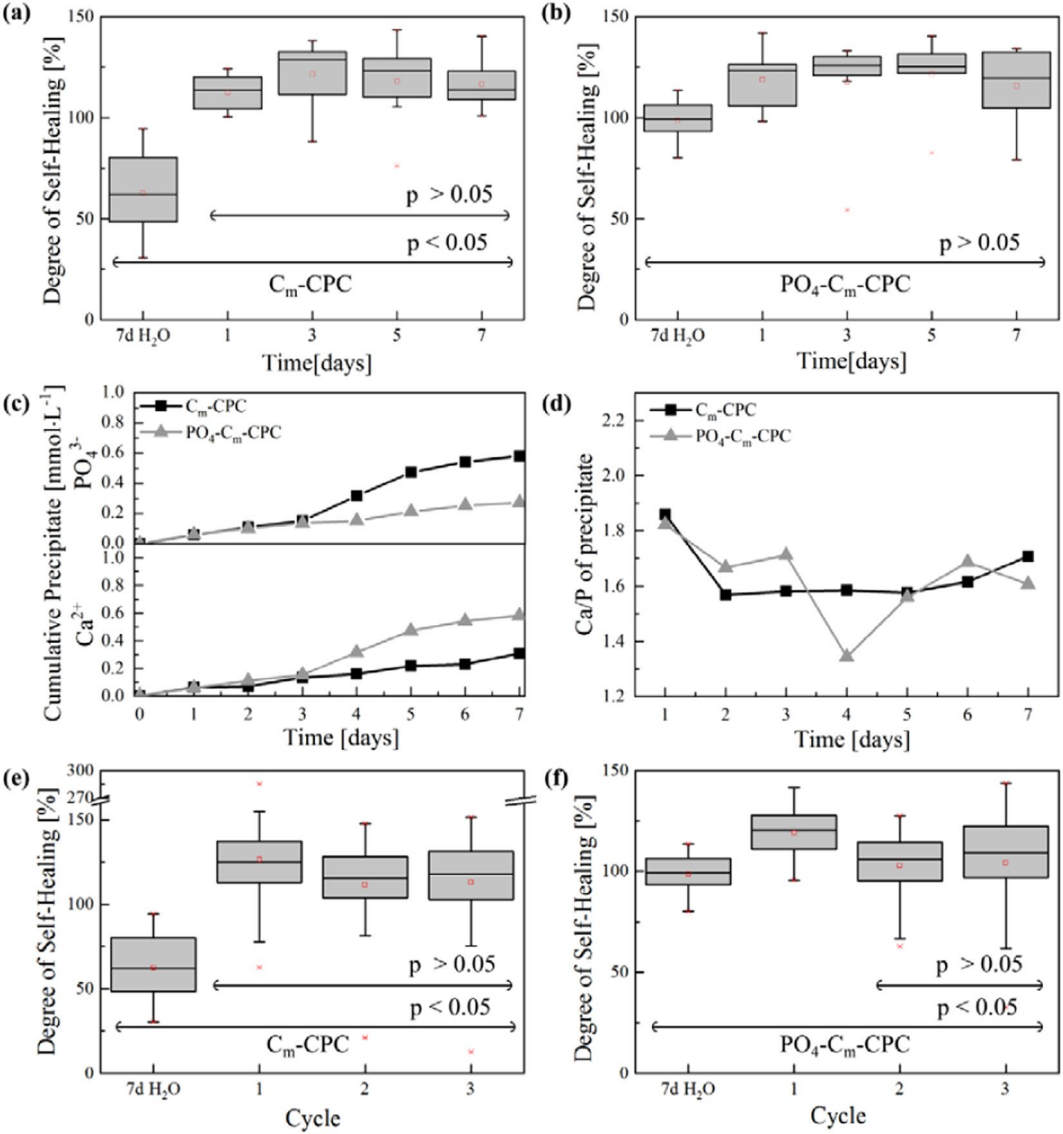
Kinetic study of C_m_-CPC and PO_4_-C_m_-CPC (**a,b**) with respect to *SHD* as a function of healing time and ICP-OES analyses of the SBF composition during healing showing (**c**) the cumulative precipitation of Ca^2+^ and PO_4_^3-^ and (**d**) the calcium-phosphor ratio. The measurement error for cumulative precipitate is about 1%. Repeatability of self-healing for C_m_-CPC and PO_4_-C_m_-CPC (**e,f**) shows *SHD* for different cycles of self-healing. The self-healing was tested in SBF. Results from ANOVA are indicated with p </> 0.05.

Figure 8 illustrates the healing kinetics and repeatability for C_m_-CPC and PO_4_-C_m_-CPC. Measurements of the mechanical recovery in dependence of time revealed the beginning of healing at day one with statistical significance (p < 0.05) (Fig. 8a,b). Hereby, only negligible differences were observed in the average *SHD* over the time period of 7 days (p > 0.05). Analyses of the amount of precipitate from the media are shown in the Figure 8c and reveal a precipitation over the whole time period. Slight differences between C_m_-CPC and PO_4_-C_m_-CPC are noted by the increased concentration of PO_4_^3-^ in solution for PO_4_-C_m_-CPC. Fig. 8e,f demonstrate the repeatability of self-healing for C_m_-CPC and PO_4_-C_m_-CPC. Self-healing was proven for C_m_-CPC over three cycles without significant efficiency loss (p > 0.05), whereas a significant decrease in *SHD* from cycle 1 to 2 was observed for PO_4_-C_m_-CPC (p < 0.05), whereas to cycle 3 no further decrease was detectable (p < 0.05).

**Figure 9:**
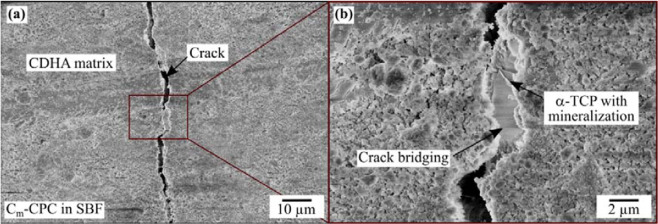
SEM of a C_m_-CPC after immersion in SBF showing incomplete filling of a crack in (**a**) overview and (**b**) detailed view with an α-TCP grain in the crack.

Figure 9 shows a crack in a C_m_-CPC during closure *via* mineralization. An overview of the crack is given in Fig. 9a, which shows mineralization and crack bridging at several points. In the detail view in Fig. 9b one particular spot is magnified, exposing a sheared α-TCP grain with mineralization at its surface. The mineralization product, similar to the previously shown results, is of porous nature and consists of needles.

## Discussion

**Figure 10:**
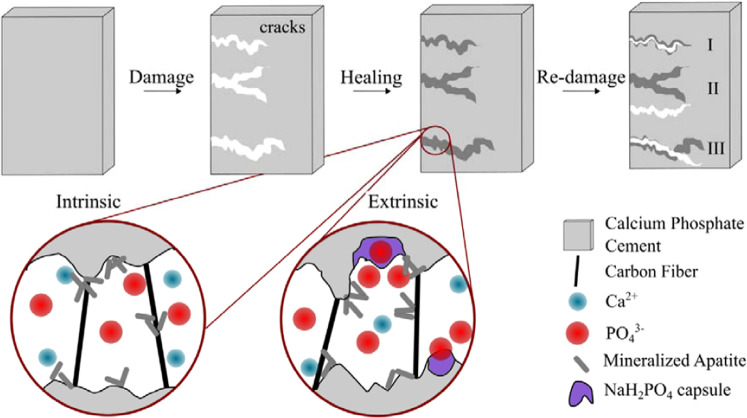
Self-healing and re-damage mechanisms of CPC with visualization of possible crack paths (I-III) during re-damage.

The self-healing capacity of damage tolerant CPC reinforced with C-fibers has been successfully demonstrated through an intrinsic as well as extrinsic mechanism. The damage tolerance results in crack stabilization, which is seen as a prerequisite to gain time for a subsequent healing process. The healing itself was achieved by the mineralization of cracks in SBF, mimicking the inorganic composition of human blood plasma. After a setting time of 7 days for all samples, the matrix mainly consisted of CDHA with a low content of α-TCP (Fig. 3). The addition of both C-fibers and capsules led to a reduction of the α-TCP amount (Table 2). The use of C-fiber reinforcement with both, untreated (C-CPC) and *aqua regia*/CaCl_2_ treated fibers (C_m_-CPC), respectively, lead to a significant increase of strength^35^. Changes during the setting of the cement, which are shown both in setting times and in phase analyses of the cements, as well as negatively charged surface groups on the fiber led to strong fiber-matrix interactions, which resulted in an altered fracture behavior^35^. This finally contributed to fulfilling our first requirement of effective bridging and thus stabilization of cracks. The additional incorporation of capsule system, consisting of polymer coated NaH_2_PO_4_ salt crystals (PO_4_-C-CPC and PO_4_-C_m_-CPC), lead to a further increase of strength, due to changes in the cement setting kinetics (Table 3). Since some of the NaH_2_PO_4_ salt is exposed during mixing due to rupture of the coating, both, the subsequently reduced pH of the paste and the oversaturation with H_2_PO_4_^-^ most likely accelerate the nucleation of calcium phosphate phases such as brushite, octacalcium phosphate or amorphous calcium phosphate, which transform into CDHA when the pH increases to a physiological level. As a result, the CPC matrix consists of smaller crystals (Fig. 4). It is well known that the crystal size has a strong influence on the mechanical properties, whereby smaller crystals can result in higher strength due to their more effective energy dispersion^45^. Beyond, it was shown that the C-fiber reinforcement causes multiple cracking. While in a standard CPC one crack would open and propagate catastrophically, here several cracks are opened, and the energy is dissipated by fiber-matrix interactions, resulting in steady state crack propagation with narrow crack openings below 10 µm (Fig. 5). This damage tolerance due to fiber incorporation can also be seen by the drastic increase of *WOF5* (Table 3). Whereas pure CPC shows a negligible *WOF5* of 0.02 kJ∙mm^−2^, the fiber incorporation of untreated or chemically modified fibers causes an increase of *WOF5* by a factor of 35 and 95, respectively^35^. Beyond the effect of fiber reinforcement, also the influence of the H_2_PO_4_^-^-supply was investigated. As mentioned earlier, partial release of H_2_PO_4_^-^ results in a smaller crystal size of the CDHA matrix and therefore a more effective crystal interlocking and energy dissipation. Consequently, also PO_4_-C_m_-CPC shows multiple cracking with narrow openings. This effect is not only showing in enhanced strength, but also in a *WOF5* of 1.7 kJ∙mm^−2^. As a result of these cracking processes all of the investigated CPC systems are damage tolerant. Besides the damage tolerance, crack stabilization at narrow openings is also believed to be essential for a successful mineralization of the cracks for self-healing of the CPCs, since mineralization processes are time consuming and the thickness of mineralized layers depends on time^46^. On a bioactive surface exposed to SBF mineralization is observed within a few days, as schematically illustrated in the detail views of Fig. 10. Hereby, a charged surface attracts oppositely charged ions from solution and the nucleation of a calcium phosphate is initiated^47,48^. In the further process, a biomimetic apatite layer consisting of small platelet shaped crystals is formed^38^. In the present system, the CPC matrix that consists of CDHA crystals acts as bioactive surface, attracting Ca^2+^ and PO_4_^3-^ ions from solution^49^. As a result, biomimetic apatite nucleates and grows (Fig. 7a,b). Beyond that, the chemically treated fibers can act as additional nucleation sites, either due to their negative surface charge or, due to matrix material present on the fibers surface after fracture. Hereby, the microstructure of the matrix might also influence the bioactivity, since with a higher specific surface also more nucleation sites are present^50^. In addition to the bioactivity of the crack surfaces and the fibers, however, the ion supply, especially PO_4_^3-^, also limits the mineralization rate^51^. A second pathway to mineralization is observed in the case of additional NaH_2_PO_4_ capsules in the composite. Here, the crack most likely induces mechanical rupture of the polymer coating and a subsequent dissolution of the salt. As a result, a local H_2_PO_4_^-^ oversaturation in the direct surrounding of the capsule occurs, causing the precipitation of a CaP and the storage at physiological pH in SBF or water leads to the formation of apatite. Examination of the surface ratio of capsules on the fracture surface of CPC revealed a content of 7%, which is exposed and presumably ruptured due to crack propagation. Therefore, a capsule-based extrinsic initiation of mineralization is most probable. The mineralization independent on whether it is initiated by active crack flanks, fiber coatings or a capsule induced oversaturation, causes in a first instance a shortening and narrowing of the crack, which overall reduces the defect size. Additionally, due to mineralization sites along the crack surface such as fibers or capsules, a bridging effect can occur. In this process, the precipitation closes the crack at certain points, which on the one hand stabilizes the crack and on the other hand splits the crack (Fig. 9). This shielding can be expected to reduce the size of defects, which according to Griffith directly affects the strength and thus leads to the restorage of the mechanical properties (Fig. 7c,d)^52^. Additionally, beyond the relation of the maximum strengths in first and second loading, also similarities in their slopes indicate the self-healing of the CPCs (Fig. 6). The exemplary curves show a steep slope and isolated drops for samples during the first loading. Hereby the drops are caused by crack opening followed by an increase due to the stabilization by fibers. Upon second loading, for samples stored in water the slope and maximum strength are dramatically decreased, whereas after storage in SBF both, the maximum strength as well as the slope are very similar to the first loading. The absence of individual steps may indicate that similar cracks are reopened. Since the slope and strength are nevertheless similar, a possible conclusion might be, that although crack opening is facilitated due to changes in the sample surface, the crack propagation through the materials volume is similar to undamaged CPC. In addition to crack healing, some* SHD* have averages values exceeding 100%, which would correspond to a strengthening of the cement. The strength of ceramics is determined by the distribution of defects and their size. The smaller and less defects are present, the stronger is the material, since flaws initiate the propagation of cracks^53,54^. Thus, the presented healing mechanism is not limited to newly formed defects by e.g. loading, but also affects pre-existing ones. As a result, the overall defect size is reduced, resulting in higher strength. This also affects the crack formation upon second loading of a healed sample. Several events are conceivable, as schematically illustrated in Fig. 10. Either (I) the initial crack is re-opened and the propagation takes place along the same path, or (II) new cracks at different positions are opened. Beyond, (III) variations from the crack course might occur. Hereby, the change of crack geometry by mineralization is decisive. Mineralization itself depends amongst others on the ion-supply as well as on the presence of nucleation sites. These parameters are influenced by the porosity and the direct surrounding of the defect. In the case of cracks, the perfusion is ensured and even supported by capillary forces, whereas in the case of pre-existing defects the porosity is determining and perfusion can be impeded. Hence, the successful healing of pre-existing or newly formed defects strongly depends on the direct surrounding and which mechanism of re-damage is activated. If a crack is not completely filled, or no other defects are present, the re-opening of the crack is most likely. On the contrary, a fully mineralized crack increases the probability for the formation of new cracks starting at different defects. Finally, inhomogeneities in the filling might lead to a change of the crack pathway. In particular, this is likely, if the fiber stabilization leads to variations of the crack opening during its propagation.

The mineralization itself is a continuous process, that was investigated kinetically for C_m_-CPC and PO_4_-C_m_-CPC in terms of mechanical restoration and time dependent analyses of the healing media (Fig. 8). Here, the mechanical characterization revealed that even for a low amount of precipitate from solution, the healing occurred instantly. Since, the defect size is essential for crack propagation, even a low degree of mineralization might have an enormous impact on the defect size by shielding of cracks and consequently on the mechanical properties. In the course of the healing process, the degree of mineralization from solution increases. After one day a fast decline of Ca^2+^ concentration is observed, resulting in a high calcium-phosphor ratio (Ca/P) ratio of 1.9. This is in agreement with the described mineralization mechanism of biomimetic apatite on synthetic apatite^55^. Besides, less time is observed in the case of the PO_4_-C_m_-CPC. This indicates, that initially the capsule material is dissolved and precipitated, before ions are consumed from the SBF. However, the release and adsorption processes of the CPC matrix material impede the interpretation of the precipitation processes from the SBF, which makes it difficult to interpret the ICP-OES results. Since the matrix material consists of CDHA it has an inherent driving force to attract Ca^2+^ from solution due to the presence of calcium voids in the crystal lattice. On the contrary, it was shown in a previous study, that CDHA cements release phosphate ions^35^. This could also be responsible for the higher Ca/P ratio, which is typically around 1.5 for biomimetic apatite^55^. Nevertheless, since the ICP results are in accordance with the mechanical characterization of healing in water for PO_4_-C-CPC and PO_4_-C_m_-CPC, participation of capsule material in the mineralization process can be assumed. It is believed that the dissolution of the encapsulated salt in combination with a setting of unreacted powder present in the CPC or ion release from the matrix, initiates mineralization and hence healing. The repeatability of the self-healing was tested for both C_m_-CPC and PO_4_-C_m_-CPC with healing periods of 5 days. Self-healing over three cycles is verifiable with *SHD* exceeding 100% for both, extrinsic and intrinsic healing (Fig. 8e,f). Since healing is based on mineralization from SBF and inherent crack stabilization, the healing process is efficient as long as the ion supply is assured. This distinguishes our extrinsic self-healing mechanism from most systems known in literature. Usually, extrinsic healing is only possible once, because no healing agent is available for further cycles^5^. In our presented case, intrinsic self-healing dominates, when the healing agent is consumed. This assumption is supported by a slightly reduced healing efficiency from the first to the second cycle from 119% to 103% with statistical significance (p < 0.05), while no further change could be detected in the third cycle.

However, *in vivo* some significant differences to our *in vitro* model are expected. First of all, instead of the static presence of the liquid, a perfusion with human blood is expected, enhancing the ion supply and accelerating the mineralization rate^56^. Nevertheless, besides the inorganic components of SBF, also organic ones e.g. proteins, are present *in vivo* which can decelerate the formation of apatite^57^. Therefore, we suspect, that the kinetic *in vivo* will differ from the *in vitro* model. Despite their inhibitory effect, the organic building blocks finally lead to a crack filling of high compositional similarity to human bone. Bone itself consists of apatite and collagen, which profits from its unique hierarchical structure that leads to its outstanding mechanical properties^58^. Therefore, the newly formed material in cracks should perform with superior mechanical properties compared to the man-made implant material. Beyond, CPCs are resorbed *in vivo* and transformed to new bone^43,59^. During this remodeling process the C-fibers are expected to remain in place and the new bone will be formed around them. Since apatite is only poorly solvable *in vivo*, the remodeling process will take months to years depending on implant size, making the self-healing properties so essential. Due to the small crack openings, associated problems with the ingrowth of blood vessels might occur *in vivo*, which could limit the healing in comparison to *in vitro* conditions^60^. Nevertheless, it is due to the small crack opening that CPCs show damage tolerance, which we suspect to be sufficient for stabilizing the cracks under cyclic loading associated with *in vivo* demands. The increased strength due to the incorporation of both untreated and treated fibers results in sufficient mechanical strength for moderate load bearing applications, e.g. in vertebroplasty. Here, Wilke *et al.* measured compressive loads *in vivo* in the spine in the range of 0.1–2.3 MPa, which is lower than the compressive strength of the α-TCP cement system used in our study^61,62^.

## Conclusion

Self-healing of CPC with respect to the restoration of mechanical properties was achieved, when two key factors, damage tolerance and ion supply are realized simultaneously. Investigation of the crack closing took place for both, extrinsic and intrinsic healing. Intrinsic self-healing requires the presence of calcium and phosphate ions in solution and is therefore solely observed in SBF. Here, bioactive crack area, as well as functionalized C-fibers promote the nucleation of apatite. An extrinsic healing by the NaH_2_PO_4_ capsules is assumed, since a healing of cements with capsules is also observed in demineralized water. The release of H_2_PO_4_ from the capsules leads to local supersaturation and thus to mineralization within the cracks. In both cases the mineralization leads to effective change of crack opening and even crack closing, whereby mechanical properties are restored. Due to self-healing over three cycles, a high repeatability can be assumed. This offers new fields of application for the class of CPC, also potentially in load-bearing areas.
